# 52 Procedures in 52 Weeks: An Innovative Curriculum for Emergency Medicine Residents

**DOI:** 10.5811/westjem.2016.9.31254

**Published:** 2016-11-21

**Authors:** Ryan Walsh, John Bass, Chad Gorbatkin, Jason Bothwell

**Affiliations:** Madigan Army Medical Center, Department of Emergency Medicine, Tacoma, Washington

## BACKGROUND

The specialty of emergency medicine (EM) requires that providers are as competent in rare procedures as they are in common ones.^1^ Emergency physicians (EPs) need to be able to perform an array of procedures, many of them life-saving, often on very short notice. The Accreditation Council for Graduate Medical Education (ACGME) has identified 18 “key procedures” as training requirements for EM residency programs.^2^,^3^ However, the list of requisite procedures does not even approach the number of procedures that are encountered in the scope of EM practice. For example, lateral canthotomy, escharotomy, and resuscitative thoracotomy are potential eye, limb, and life-saving procedures. All are infrequently encountered, within the scope of an EP’s practice, and yet not required by the ACGME. This creates a challenge for EM educators, who remain charged with graduating competent physicians who have the mental and technical expertise to perform such procedures.^4^,^5^ Another challenge faced when teaching and, more importantly, performing rare procedures is logistics. The required equipment is often expensive, stored in small quantities, and sometimes difficult to find. Equipment setup and application is a perishable skill, and rehearsal is essential to ensure the success of the procedure when it is finally needed.

## OBJECTIVES

Our objective was to implement a high-yield weekly training session that effectively teaches important emergency department procedures. We wanted our residents to not only become competent in the mental and technical aspects of performing the procedure, but also the logistics of finding and assembling the required materials within our own department. Additionally, when it came to pre-packaged and sterile surgical sets, we wanted our residents to learn what was contained within the sets so that in an emergent scenario they would already be familiar with the contents. Lastly, we wanted to use a method of instruction that best used our available resources outside of the traditional classroom.

## CURRICULAR DESIGN

After polling all the residents and attendings at our program, we compiled a list of 52 EM procedures. These ranged from emergent life-saving procedures such as cricothyroidotomy and pediatric jet ventilation to ring removal and nerve blocks. Once the list was compiled, we searched the Internet and located high-quality instructional videos for each procedure (Supplement 1)*.* We then published this list of 52 procedural videos to our residency website and implemented it into our morning report curriculum, which historically consisted of an oral boards case or a simulation case during morning shift change.

For this “procedure morning report,” we have a second-year EM resident paired up with an attending, teaching the assigned procedure every Friday. The instructors guide learners through the assigned procedure by first showing the instructional video, and then pairing this with hands-on training using a variety of simulated and tissue models, home-made training devices, or other necessary equipment. By design, the training takes place in our ED rather than in the classroom. Instructors demonstrate the location of all equipment, and actually open the kits to show the learners all of the component parts and how to use them. Participants are the off-going night shift and the oncoming day shift, for a total of eight EM residents, plus any rotating residents and students. Nurses also participate when the procedure may require their assistance. The training typically lasts approximately 30 minutes, and always includes hands-on participation by the learners.

## IMPACT/EFFECTIVENESS

After two years of implementation, we surveyed the current 36 EM residents regarding their experiences with the curriculum. Seventy-five percent of our residents (27/36) responded to the survey. Eighty-five percent (23/27) of respondents found the published instructional videos easily accessible and “very helpful.” All respondents reported increased competence and confidence following the instruction. Among second- and third-year participants, all of whom had been instructors and learners, 80% found the role of instructor to be “very helpful in skill mastery.” While not specifically included in our survey, other noted benefits include a teaching role for second-year residents and the integration of nursing in our curriculum. With over two years of experience now, we have found that this has been a worthwhile addition to our curriculum that can be easily implemented in other EM residencies.

One limitation of this curriculum is the cost of supplies. To mitigate this in our department, we try to use expired products whenever possible and save training sets for future use. Another limitation is that the effectiveness of the curriculum is based off residents’ subjective feedback. Future research can evaluate procedural performance before and after implementation.

## Supplementary Information


