# Supplementary material for: 52 Procedures in 52 Weeks: An Innovative Curriculum for Emergency Medicine Residents
Source: West J Emerg Med. 2016 Nov 21;18(1):12–3. doi: 10.5811/westjem.2016.9.31254 (PMC5226743; doi:10.5811/westjem.2016.9.31254)
Supplement: Supplementary file 1 [file wjem-18-12-s001.pdf]

Procedure

Distal radius fracture, boxer's fracture/reduction and hematoma block
Shoulder dislocation reduction techniques
Burr Holes
iLMA usage
Mandibular dislocation reduction
Knee arthrocentesis
Tendon Repair
Suprapubic catheter
Perimortem Csxn

Digital intubation/ Retrograde Intubation
Needle Aspiration of Pneumothorax
Pelvic binding
Sager Traction splint
Cricothyroidotomy/ bougie assisted cricothyroidotomy
posterior hip dislocation/ elbow dislocation
Lateral Canthotomy
Ear Hematoma Treatment
Peds failed airway (LMA, jet ventilation)
Posterior tibial nerve block (ultrasound)
Cervical neck injections for headaches
ED repair of dialysis graft bleed
Pneumothorax: Pig Tail Catheter Insertion
Ring Removal

Thoracentesis
Peritonsilar abscess I&D and nail trephination
Ultrasound guided shoulder joint injection
Bougie tips and tricks
Chest tubes / Pleurovac management

Anterior and posterior epistaxis management

Transvenous pacemaker placement
Priapism/ dorsal nerve block
Amputated toe repair
Infant spinal tap
Foreign body in nose and ear removal
Subacromial bursa injection/ IO mistakes
Ingrown toenail/ Felon management
Paracentesis
Fish hook removal

NP scope
Awake intubation
Forearm nerve blocks (ultrasound)
Epley Maneuver

Bartholin cyst/abscess I&D

Dental blocks
ED thoracotomy
Steps in laryngoscopy
arterial line (blind & US guided)
Perircardiocentesis (blind & US guided)
Blakemore Tube placement
Thrombosed hemorrhoid excision
External jugular vein cannulation
vent for preoxygenation;

Link

https://www.youtube.com/watch?v=Q1fE_rhFUXo&index=6&list=PL5e1rUuEhQ3qH5i95d2CXEH-GdwZ8RkOO
https://www.youtube.com/watch?v=HtOnreM7heg
https://www.youtube.com/watch?v=fJUQf1GaQkg
https://www.youtube.com/watch?v=Apt7jlnQx2I
https://www.youtube.com/watch?v=aGknbegDkl4
https://www.youtube.com/watch?v=ElLcgeGQWaw
https://www.youtube.com/watch?v=qKTYV7x1nHc
https://www.youtube.com/watch?v=3C-dBru73-E
https://www.youtube.com/watch?v=Y0jQt2OVQtk

https://www.youtube.com/watch?v=Yb1sGsUAZ-E
http://www.youtube.com/watch?v=j_UGBS-Kp2I
https://www.youtube.com/watch?v=Sp_FkLTyE9E
https://www.youtube.com/watch?v=c-3bptUGXkw
http://emcrit.org/procedures/cricothyrotomy/
http://www.youtube.com/watch?v=lQMWaFX-MeQ
http://www.youtube.com/watch?v=bUAagMd_Q8A
http://www.youtube.com/watch?v=ZZtT-E3NGSY
http://www.youtube.com/watch?v=XMtPg6wkdKI
http://www.youtube.com/watch?v=ak6QA3Zb7vE
http://www.youtube.com/watch?v=0to5wzftpnM
http://www.youtube.com/watch?v=toFiGSfesZk
http://www.youtube.com/watch?v=xsB9MkuCQE4
http://www.youtube.com/watch?v=DxoAbK5Pc6w
https://www.youtube.com/watch?v=AlCCsUUf7MA&list=PL9UKTUFtRDcNq4--Vf2NYfUANEyObfeNm
http://www.youtube.com/watch?v=bLEGfl9WE30
http://www.youtube.com/watch?v=siGzMvakY8s
http://lifeinthefastlane.com/education/ccc/bougie/
https://www.youtube.com/watch?v=f_JWzLiUNhE
http://www.emergentprocedures.com/Emergent_Procedure_Instructional_Collaboration/Anterior_Epistaxis.html
http://www.emergentprocedures.com/Emergent_Procedure_Instructional_Collaboration/Transvenous_Pacemaker.html
http://www.youtube.com/watch?v=KWf5MAobWoM
http://www.youtube.com/watch?v=hmmov4CK1uk
http://www.youtube.com/watch?v=jE1ZKNbwSIg
http://www.youtube.com/watch?v=QBcvcn5C0ic
http://www.youtube.com/watch?v=n0IcrLmpIsY
http://www.youtube.com/watch?v=t6J_pVEIlUQ
https://www.youtube.com/watch?v=KVpwXK7cvzQ
https://www.youtube.com/watch?v=YyymouV8ulU

http://www.youtube.com/watch?v=Q6Uz1qYwGl4
http://vimeo.com/18057952
http://www.youtube.com/watch?v=8-n5bnT9uog
http://www.youtube.com/watch?v=59EIKztATiw
http://www.operationalmedicine.org/Videos/Bartholin1.mpg
https://sites.google.com/site/emprocedures/facial-and-dental-nerve-blocks
http://www.youtube.com/watch?v=8BlPxQI2C90
http://vimeo.com/17542057
http://www.youtube.com/watch?v=YOxyssqqYNE
http://www.youtube.com/watch?v=M4vHEr25yFk
https://www.youtube.com/watch?v=EGcEiUIY9vY
http://www.youtube.com/watch?v=wdVkcWsUHoc
https://www.youtube.com/watch?v=aGIwUGEgSZA
http://vimeo.com/35483346

Secondary Link

https://www.youtube.com/watch?v=EhJ7kpurKnk

https://www.youtube.com/watch?v=jD3JTOaS2-0
https://www.youtube.com/watch?v=5CYqmvFIAzg

http://www.youtube.com/watch?v=Hm1xxE318fU&list=UUMnjOoVTC5
ef7eIUcurTXDQ&index=32

https://www.youtube.com/watch?v=Omg79Ced6s0

http://emcrit.org/procedures/bougie-aided-cric/
http://lifeinthefastlane.com/2013/08/love-the-therapeutic-clunk/

http://www.youtube.com/watch?v=OzAbvPbpO18
http://www.youtube.com/watch?v=jk19A8v7TtA
http://www.youtube.com/watch?v=jT9xsw_ElhE

http://www.youtube.com/watch?v=6ThpUpgjSiM
https://www.youtube.com/watch?v=I5W27zV-dwI

http://www.youtube.com/watch?v=tIo7qASsUn4
http://www.emergentprocedures.com/Emergent_Procedure_Instructio
nal_Collaboration/Posterior_Epistaxis.html

http://www.youtube.com/watch?v=c260y8ZRUuk
https://www.youtube.com/watch?v=3p0qEfISggs

http://www.youtube.com/watch?v=ARQhqeHfzOY
http://www.youtube.com/watch?v=YXfyL8kvFTg
https://www.youtube.com/watch?v=BuP4UGfutWk
http://www.youtube.com/watch?v=zyvHGmigvD0

http://www.bing.com/videos/search?q=nasopharyngeal+scope&view=detail&mid=BE8C77CB57BF5958690CBE8C77CB57BF5958690C&first=0&FORM=NVPFVR

http://www.youtube.com/watch?v=6NKkzs9FA5I

http://academiclifeinem.com/trick-of-the-trade-alternative-to-word-catheter-for-bartholin-abscess/
https://www.youtube.com/watch?v=MWk2vbUo2jQ&list=UU_yjveGdyx6mqqHkHaD-_bg
http://www.youtube.com/watch?v=A57ZB_J4FuY

http://www.youtube.com/watch?v=Vt7ONGDeP3w
http://www.youtube.com/watch?v=sy6pKUJzPOk
https://www.youtube.com/watch?v=imFCMWeWDpU

3rd Link

https://www.youtube.com/watch?v=6fXYHhb_P0E

https://www.youtube.com/watch?v=11Wn7tXDzQA

http://www.youtube.com/watch?v=nrxuZwFpigI

http://www.youtube.com/watch?v=PoGlBqPvJ-Y

http://www.youtube.com/watch?v=GPAXS7FyQHQ
https://www.youtube.com/watch?v=48jOPHs-L9w

https://www.youtube.com/watch?v=IGASYCevWag

https://www.youtube.com/watch?v=muTxACEsXx0
